# Assessment of knowledge and practice of menstrual hygiene among high school girls in Western Ethiopia

**DOI:** 10.1186/s12905-015-0245-7

**Published:** 2015-10-14

**Authors:** Shivaleela P. Upashe, Tesfalidet Tekelab, Jalane Mekonnen

**Affiliations:** College of Medical and Health sciences, Wollega University, P.O. Box: 395, Nekemte, Oromia Ethiopia

**Keywords:** Knowledge, Menstruation, Practice, Sanitary pads, Hygiene

## Abstract

**Background:**

The issue of menstrual hygiene is inadequately acknowledged and has not received proper attention. Use of sanitary pads and washing the genital area are essential practices to keep the menstrual hygiene. Unhygienic menstrual practices can affect the health of the girls and there is an increased vulnerability to reproductive tract infections and pelvic inflammatory diseases and other complications. Therefore, the objective of this study was to assess the knowledge and practice of menstrual hygiene among high school girls at Nekemte town, Oromia region, Western Ethiopia.

**Method:**

A school based cross-sectional study design was employed in Nekemte Town, Western Ethiopia. A multi stage sampling technique was used to select 828 female high school students. Data collection was carried out from May 04 to May 30, 2014 using a pre- tested structured questionnaire. The data were entered into a computer using Epi-info version 3.5.1 and then exported to SPSS for Windows version 20.0 for analysis. Bivariate and multivariate logistic regression analysis was done at 95 % confidence interval.

**Results:**

In this study, 504 (60.9 %) and 330 (39.9 %) respondents had good knowledge and practice of menstrual hygiene respectively. The findings of the study showed a significant positive association between good knowledge of menstruation and educational status of mothers (AOR = 1.51, 95 % CI = 1.02 – 2.22), having radio/TV (AOR = 2.42, 95 % CI: 1.64 – 3.56). Educational status of the mother (AOR = 2.03, 95 % CI = 1.38 – 2.97) and earning permanent pocket money from parents (AOR = 2.73, 95 % CI = 1.76 – 4.26) revealed significant positive association with good practice of menstrual hygiene.

**Conclusions:**

The findings showed that the knowledge and practice of menstrual hygiene is low. Awareness regarding the need for information about good menstrual practices is very important. So, health education program should be setup to create awareness and practice of good menstrual hygiene.

**Electronic supplementary material:**

The online version of this article (doi:10.1186/s12905-015-0245-7) contains supplementary material, which is available to authorized users.

## Background

Adolescent girls constitute a vulnerable group not only with respect to their social status but also in relation to their health. In this regard, menstruation is regarded unclean or dirty in society [1]. The issue of menstrual hygiene is inadequately acknowledged and has not received proper attention [2]. Good hygienic practices, such as use of sanitary pads and adequate washing of the genital areas, are essential during menstruation period. Women and girls of reproductive age need access to clean and soft absorbent sanitary products which in the long run protect their health from various infections [3]. To this effect, the practice of good menstrual hygiene reduces the incidence of reproductive tract infection (RTI). Thus, the consequences of RTIs are severe and may result in significant negative impact to a woman’s health including chronic pelvic pain, dysmenorrhea (painful periods) and in severe cases infertility. Reproductive tract infections, which have become a silent epidemic that devastates women’s lives is closely related to poor menstrual hygiene [1, 4].

Every year approximately 10 % of women worldwide are exposed to genital infections including urinary tract infections and bacterial vaginosis, and 75 % of women have a history of a genital infection. Specifically, the common risk factors for vaginal infections include pregnancy and poor hygiene (both perineal and menstrual hygiene) [5].

Studies in Africa have found out the use of sanitary pads as low as 18 % amongst Tanzanian women with the remainder using cloth or toilet paper [6]. Studies of Nigerian schoolgirls have found between 31 and 56 % using toilet tissue or cloth to absorb their menstrual blood as opposed to menstrual pads [7, 8].

A study conducted in Ethiopia showed that, though, most (92 %) students were aware of menstruation before menarche, their utilization of sanitary napkins was low at 37.6 % and a significant proportion, 62.4 % were using rags and pieces of cloth [9]. Eleven percent of girls in Ethiopia change their menstrual cloths once a day [10].

Most girls in Ethiopia are at risk of getting genitourinary tract infections due to their unhygienic practices during their menstruation period which may lead to further complication if left untreated [11].

Therefore, this study was aimed to assess the knowledge and practice of menstrual hygiene among high school girls in Western Ethiopia. The information obtained from this study will be used by policy makers and stakeholders to identify the awareness and practice of menstrual hygiene so as to provide information about menstruation and menstrual hygiene for high school girls in the study area.

## Methods

### Study design, setting and participants

School based cross- sectional study was employed from May 04 to May 30, 2014 among high school girl students in Nekemte Town, Oromia Region, Western Ethiopia. Nekemte is the capital of East Wollega Zone (Province) located at 331 km from Addis Ababa. The total population of the town is estimated to be 75,219 of which 38,385 (51 %) was females. There are ten high schools (4 governmental and 6 non-governmental) in Nekemte town. In this town the total number of students enrolled for 9^th^ and 10^th^ grade levels were 5548 for the academic year 2013–2014 out which 2762 were male and 2756 were female [12, 13]. Girls studying in 9^th^ and 10^th^ grade were 1400 and 1392 respectively. The girls who attained menarche were included for the study. Girls with visual impairment, evening class students and those who were critically ill and incapable to provide informed consent were excluded from the study.

### Sample size and sampling procedures

The sample size was determined using a formula for estimation of single population proportion with the assumption of 95 % confidence interval, 5 % margin of error, and prevalence of knowledge about menstruation at 51.36 % [14], and design effect of 2. To compensate for the non-response rate, 10 % of the determined sample was added up on the calculated sample size and the final sample size was found to be 845.

The sampling procedure started by stratifying the schools into two categories, governmental and non-governmental. The selection of the schools was done randomly. Each school was further stratified by their sections. For selection of representative numbers of students, the ratio of students in the respective types was considered. The sample size was allocated for the schools using population proportion to the sample for each selected school, size being the number of students in each high school (9^th^ and 10^th^ grade). Finally, proportional number of participants (students) was selected by simple random sampling technique. The sampling frame was obtained from the student registration books of the respective schools.

### Data collection procedures

To collect data self-administered questionnaires were employed. After reviewing relevant literature questionnaires were adapted and modified [14–19]. The questionnaire was prepared in English language and translated into Afan Oromo, the regional language and then translated back to English by other people who are proficient in both languages to maintain the consistency and content of the questionnaire. Six girls with high- school education were recruited as data collectors. They were given a day training to familiarize them with the objective and relevance of the study, confidentiality of information, participants’ rights and informed consent. Three graduate colleagues from health supervised the data collection procedures. Their supervision, involved reviewing all questionnaires at the end of every day, and morning meetings with the data collectors to discuss any problems they encountered during data collection and provide timely remedy.

Students’ menstrual knowledge score was calculated out of the 7 knowledge specific questions (Table 2). Each correct response earned one point, whereas any wrong or don’t know response attracted no mark and thus the sum score of knowledge was calculated (7 points). Accordingly, the mean score of menstrual knowledge (4.8 ± 1.67) was used to decide the cutoffs of the rank. Good knowledge of menstruation and menstrual hygiene was given to those respondents who scored 5–7 points and Poor Knowledge of menstruation and menstrual hygiene was given to those respondents who scored 0–4 points.

Students’ practice of menstrual hygiene score was calculated out of the practice specific questions (Table 2). Each correct response earned one point, whereas any wrong or don’t know response attracted no mark. In here, the sum score of practice was calculated (10 points). Where, the mean score of practice of menstrual hygiene (5.1 ± 1.57) was used to decide the cutoffs of the rank. Good practice of menstrual hygiene was given to those respondents who scored 6–10 points and Poor practice of menstrual hygiene was given to those respondents who scored 0–5 points.

### Data processing and statistical analysis

Each completed questionnaires was coded on pre-arranged coding sheet by the principal investigator to minimize errors. Data were cleaned and entered into a computer using Epi-info Window version 3.5.1 statistical program. Then the data were exported to SPSS Windows version 20.0 for analysis. The descriptive analysis including proportions, percentages, frequency distribution and measures of central tendency was done.

Initially, bivariate analysis was performed between dependent variable (Knowledge and practice of menstrual hygiene) and each of the independent variables (Socio-demographic variables), one at a time. Their odds ratios (OR) at 95 % confidence intervals (CI) and *P*-values were obtained, to identify important candidate variables for multivariate analysis. All variables found to be significant at bivariate level (at *P*-value < 0.05) were entered in to multivariate analysis using a logistic regression model in order to control for confounding factors.

### Ethical considerations

Ethical clearance and permission was obtained from Wollega University Institutional Research Review Board. Permission was secured from each High School through a formal letter. School directors and directresses were briefed on the relevance and objectives of the study. The purpose of the study was explained to the students and written informed consent was obtained from each participant. For those students who were under the age of consent, informed verbal consent was obtained from their parents and assent from the students. Confidentiality of information was maintained by omitting any personal identifier from the questionnaire. Students were informed of their full right to skip or ignore any question or withdraw from their participation at any stage.

## Results

### Socio-demographic characteristics of the respondents

A total of 828 high school girls were participated making a response rate of 98 %. Almost two third of (65.1 %) were in the age group less than or equal to 16 years with median age of 16 years. The majority (92.9 %) of the respondents were from the Oromo ethnic group. Half of the respondents (50.7 %) were Ethiopian Protestants. Two hundred thirty eight (29 %) of the respondents’ father completed college and above. One hundred seventy eight (21.6 %) of the respondents’ mother can read and write. Nearly one third (32.7 %) of the mothers of the respondents were housewives and three hundred thirteen (37.8 %) of their fathers were farmers. Their family mean monthly income was Ethiopian birr (ETB) 1801. The majority (86.8 %) of the respondents didn’t get permanent pocket money form their families. Out of the total respondents six hundred ninety one (83.5 %) of their family owned radio/TV (Table 1).

**Table 1:** Socio-demographic characteristics of high school girls, Nekemte Town, Oromia region, Western Ethiopia, 2014

Variables (828)	Number (%)
Age category in years	
<= 16	539 (65.1)
>16	289 (34.9)
Grade	
Ninth	537 (64.9)
Tenth	291 (35.1)
Ethnicity	
Oromo	769 (92.9)
Amhara	28 (3.4)
Gurage	18 (2.2)
Others*	13 (1.6)
Religion	
Ethiopian Protestant	420 (50.7)
Ethiopian Orthodox	292 (35.3)
Ethiopian Catholic	68 (8.2)
Muslim	42 (5.1)
Others**	6 (0.7)
Educational status of the Father’s (*n* = 820)	
Can’t read and write	98 (12)
Can read and write	200 (24.4)
G1-4	48 (5.9)
G5-8	123 (15)
Secondary	113 (13.8)
College and above	238 (29)
Educational status Mother’s (*n* = 823)	
Can’t read and write	175 (21.3)
Can read and write	178 (21.6)
G1-4	121 (14.7)
G5-8	154 (18.7)
Secondary	97 (11.8)
College and above	98 (11.9)
Occupational status of the Mother’s (*n* = 818)	
Housewife	269 (32.7)
Student	139 (16.9)
Merchant	187 (22.7)
Employed in private organization	28 (3.4)
Governmental employee	102 (12.4)
Daily laborer	82 (10)
Others	16 (1.9)
Occupational status of the father’s (*n* = 820)	
Farmer	313 (37.8)
Government employed	278 (33.6)
Merchant	96 (11.6)
Private organization	48 (5.8)
Daily Laborer	77 (9.3)
Others****	8 (1 %)
Income (ETB) (*n* = 808)	
<800	215 (26.6)
800–1000	306 (37.9)
1001–2000	118 (114.6)
>2000	169 (20.9)
Mean	1801 ETB
Have radio/TV (*n* = 828)	
Yes	691 (83.5)
No	137 (16.5)
Earn money from the family (*n* = 828)	
Yes	109 (13.2)
No	719 (86.8)

* Tigre, Shinasha, Wolyita

** Adventist, Wakefeta

**** Driver, Waiter, Tailor

### Knowledge about menstruation and its hygiene

According to the data obtained from the participants, five hundred four (60.9 %) of the respondents had good knowledge about menstruation and its hygiene. Out of total six hundred thirty seven (76.9 %) of girls knew that menstruation was a physiological process, eighty (9.7 %) of the girls believed that it was a curse from God. Five hundred nineteen (62.9 %) knew that the cause of menstruation was hormone. More than half, five hundred four (60.9 %) of the respondents knew the origin of the menstrual blood was from the uterus. Majority six hundred fifty seven (79.3 %) knew about menstruation before attaining menstruation. Three fourth six hundred twenty two (75.1 %) of girls knew about menstrual hygiene. Five hundred twenty seven (63.6 %) knew that there was a foul smell during menstruation (Table 2).

**Table 2:** Respondents knowledge about menstrual hygiene, Nekemte Town, Oromia region, Western Ethiopia, 2014

Variables (828)	Number (%)
Menstruation	
Physiological process	637 (76.9)
Pathological process	52 (6.3)
Curse from god	80 (9.7)
Don’t know	59 (7.1)
Cause of menstruation	
Hormones	519 (62.7)
Curse of god	228 (27.5)
Caused by disease	22 (2.7)
Don’t know	59 (7.1)
Source of menstrual blood	
Uterus	504 (60.9)
Vagina	226 (27.3)
Bladder	17 (2.1)
Abdomen	33 (4.0)
Don’t know	43 (5.2)
Heard about menstruation before attaining menarche	
Yes	657 (79.3)
No	171 (20.7)
Knew about menstrual hygiene	
Yes	622 (75.1)
No	206 (24.9)
Knew that there is foul smelling during menstruation	
Yes	527 (63.6)
No	301 (36.4)
Knew that menstrual blood is unhygienic	
Yes	473 (57.1)
No	355 (42.9)
Knowledge (summary index)	
Good knowledge	504 (60.9)
Poor knowledge	324 (39.1)

**Fig. 1:**
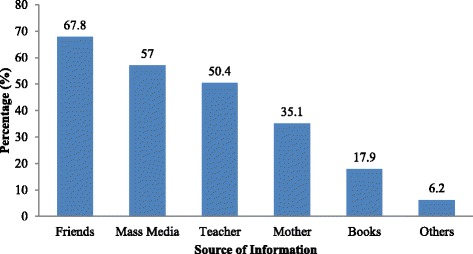
Respondents’ sources of information about menstruation in Nekemte Town, Oromia region, Western Ethiopia, 2014

The results of the study revealed that, 67.8 % of the respondents got information about menstruation from their friends, followed by mass media, teachers, from their mothers and books (Fig. 1).

Girls whose mother’s education status secondary school and above were 1.51 times more likely had good knowledge about menstruation and menstrual hygiene than their counterparts [AOR = 1.51, 95 % CI: 1.03–2.22]. Girls from families with radio and/or TV were more likely to have good knowledge about menstruation and menstrual hygiene when compared to those who had no radio/TV [AOR = 2.42, 95 % CI: 1.64 – 3.56] (Table 3).

**Table 3:** Predictors of knowledge about menstruation and menstrual hygiene among high school girls’ of Nekemte town, Oromia region, Western Ethiopia, 2014

Characteristics	Knowledge		Crude OR	Adjusted OR
	Good (%)	Poor (%)	OR (CI)	OR (CI)
Educational status of the mothers				
Below and Primary	361 (57.5 %)	267 (42.5 %)	1	1
Secondary and above	138 (70.8 %)	57 (29.2 %)	1.79 (1.27–2.53)	1.51 (1.02–2.22)*
Educational status of the father				
Below and Primary	271 (57.8 %)	198 (42.2 %)	1	1
Secondary and above	233 (66.4 %)	118 (33.6 %)	1.44 (1.08–1.92)	1.07 (0.76–1.50)
Occupation status of father’s				
Government Employed	189 (68.0 %)	89 (32.0 %)	1.55 (1.14–2.10)	1.19 (0.84–1.68)
Others	315 (57.8 %)	230 (42.2 %)	1	1
Have Radio/TV				
Yes	449 (65.0 %)	242 (35.0 %)	2.77 (1.90–4.03)	2.42 (1.64–3.56)*
No	55 (40.1 %)	82 (59.9 %)	1	1

Key = *statistically significant (*P*-value <0.05); 1 = Reference category

### Hygienic practices during menstruation

As to the data obtained, out of the total respondents, 330 (39.9 %) of the respondents had good practice on menstrual hygiene. Majority 678 (82.2 %) of girls were using absorbent material during menstruation and two third 548 (66.2 %) of girls were using commercial made sanitary pads as absorbent material during menstruation. Out of one hundred fifty seven girls who were using clothes eighty three (52.9 %) of the respondents were washing clothes with soap and water. Seventy one (45.2 %) of the respondents dried their washed clothes in sunlight. Half 430 (51.9 %) of girls change their pads or clothes three and above times per day. One hundred sixty seven (20.2 %) of the respondents were disposing their used sanitary pads in dustbin. One third 274 (33.3 %) of girls use paper to dispose the pads by wrapping. Two third 557 (67.3 %) of respondents were taking bath daily with soap during menstruation. Six hundred fifty seven (83.5 %) of the girls clean their external genitalia during menstruation with soap and water (Table 4).

**Table 4:** Respondents menstrual hygienic practices during menstruation in Nekemte Town, Oromia Region, Western Ethiopia, 2014

Parameters of practice	Number (%)
Uses absorbent materials during menstruation	678 (82.2)
Uses commercially made sanitary pad as absorbent material during menstruation	548 (66.2)
Clean clothes with soap and water	83 (52.9)
Dry cloths in sunlight	71 (45.2)
Changing pads or cloths more than three times and above during menstruation	430 (51.9)
Disposes used sanitary pads in dustbin	167 (20.2)
Uses paper to dispose the pads by wrapping	274 (33.3)
Takes bath daily with soap during menstruation	557 (67.3)
Clean external genitalia during menstruation	787 (95)
Cleans external genitalia with water and soap during menstruation	657 (83.5)
Practice (summary index)	
Good practice	330 (39.9)
Poor practice	498 (60.1)

Girls whose mother’s educational status was secondary school and above were 2 times more likely to have good practice of menstrual hygiene than their counterparts [AOR = 2.03, 95 % CI: 1.38–2.97]. Respondents whose mother’s occupations come under category of others were less likely to have good practice of menstrual hygiene than housewives [AOR = 0.66, 95 % CI: 0.47–0.91]. Girls who earn permanent pocket money from their families were nearly three times more likely to have good practice about menstrual hygiene compared to those who don’t earn permanent pocket money from their families [AOR = 2.73, 95 % CI: 1.76 – 4.26] (Table 5).

**Table 5:** Predictors of practice about menstruation and menstrual hygiene among high school girls’ of Nekemte town, Oromia region, Western Ethiopia, 2014

Characteristics	Practice		Crude OR	Adjusted OR
	Good (%)	Poor (%)	OR (CI)	OR (CI)
Educational status of the mothers				
Below and Primary	224 (35.7 %)	404 (64.3 %)	1	1
Secondary and above	102 (52.3 %)	93 (47.7 %)	1.98 (1.43–2.74)	2.03 (1.38–2.97)*
Educational status of the father				
Below and Primary	166 (35.4 %)	303 (64.6 %)	1	1
Secondary and above	164 (46.7 %)	187 (53.3 %)	1.60 (1.21– 2.12)	1.26 (0.90–1.78)
Occupational status of the mother				
House wife	127 (47.2 %)	142 (52.8 %)	1	
Others	201 (36.7 %)	347 (63.3 %)	0.65 (0.48–0.87)	0.66 (0.47– 0.91)*
Monthly income				
<800	67 (31.2 %)	148 (68.8 %)	1	1
800–1000	129 (42.2 %)	177 (57.8 %)	1.61 (1.12–2.32)	1.46 (0.99 – 2.15)
1001–2000	52 (44.1 %)	66 (55.9 %)	1.74 (1.09–2.77)	1.20 (0.73 – 1.99)
>2000	68 (40.2 %)	101(59.8 %)	0.65 (1.49–2.27)	1.24 (0.80 – 1.93)
Earn permanent pocket money from parents or relatives				
Yes	66 (60.6 %)	43 (39.4 %)	2.65 (1.76–4.00)	2.73 (1.76–4.26)*
No	264 (36.7 %)	455 (63.3 %)	1	1

Key = *statistically significant (*p*-value <0.05), 1 = Reference category

## Discussion

In this study, more than half (60.9 %) of the students had good knowledge about menstruation and menstrual hygiene. The majority (76.9 %) girl knew that menstruation was a physiological process, whereas 9.7 % them believed that it was a curse from God. The findings were higher than those in previous studies done in Ethiopia, Nigeria, and Nepal, which were 51.36 %, 4.0 %, and 40.6 %, respectively [14–16]. This difference could be due to minimal communication in families about menstruation and menstrual hygiene issues. Contrary to the findings of this study, high knowledge about menstrual hygiene was obtained in a study done in Amhara, northern Ethiopia, which was 90.7 % [17], possibly due to information provided about menstruation and menstrual hygiene by schools and families.

Almost 68 % of the respondents got information about menstruation from their friends (67.8 %), followed by mass media (57 %); teachers, mothers, and books were the main sources of menstrual information in this study. These findings are consistent with the results from studies done in Egypt and India [18, 19]. A possible explanation for this similarity may be that girls discuss menstruation and its hygiene with their friends and peers openly.

In this study, multivariable analysis showed that girls whose mothers’ educational status was secondary school and above were 1.51 times more likely to had good knowledge about menstruation and menstrual hygiene than their counterparts. A similar study done in western Nigeria showed that parental education was positively associated with girls’ menstrual knowledge [20]. This study disagrees with results obtained from a study in Sokot, Nigeria [21]. The reason could be that educated mothers may provide information about menstruation and menstrual hygiene to their daughters. Girls from educated families may discuss openly about sexual and reproductive health issues including menstruation.

The mass media play a prominent role in the dissemination of reproductive health information including menstruation and menstrual hygiene [7, 18, 22]. The knowledge level of menstrual hygiene appears to be increasing with an increase in time spent on watching TV/listening to radio [23]. Thus the finding of this study showed that the availability of mass media (Radio/ TV) at home as the highest predictor of good knowledge of menstrual hygiene. In fact, the reason might be mass media may be endorsed to the effect of technology on increasing knowledge and gaining needed information about menstrual hygiene.

In this study, three hundred thirty (39.9 %) of the respondents had good practice of menstrual hygiene. The finding of this study was lower than studies conducted in Ethiopia and North western Nigeria which were 90.9 % and 88.7 %, respectively [15, 17]. Comparatively, lower level of practice of menstrual hygiene was recorded from similar study conducted on Gujjar girls it was indicated that only 3.1 % of the study participants practice good menstrual hygiene [24]. Thus, the reason for the observed difference could be due to low awareness and communication of menstrual hygiene by Gujjar girls which affects their menstrual hygienic practice.

Educational status of the parents was important predictors of menstrual hygienic practice [20]. In the present study girls whose mothers’ educational statuses was secondary and above were two times more likely to have good practice of menstrual hygiene than their counterparts. This aligns with the studies done in Ethiopia, Lebanon, India and Nigeria [14, 21, 25, 26]. The possible explanation might be that educated mothers may have awareness on practice of menstrual hygiene and they may have provided materials for their daughters to clean their genitalia during menstruation.

This study indicated that respondents whose mothers’ occupation others were less likely to have good practice of menstrual hygiene compared to those of housewives. In contrast, a study done in Nigeria indicated that employment of mothers showed significant statistical association with respect to the practice of good menstrual hygiene [21]. The difference might be due to that majority of occupation of the respondent mothers in Nigeria were business women and civil servants. Those mothers who have work outside home may have exposure and access to information that can increase knowledge and awareness of reproductive issues including menstruation and menstrual hygienic practice.

In this study girls who earn permanent pocket money from their families were nearly three times more likely to have good practice about menstrual hygiene compared to those who don’t earn permanent pocket money from their families. Studies done in Ethiopia and South India were consistent with our study [14, 27]. This could be due to girls who get money from their parents can easily buy sanitary napkins for their menstrual hygiene.

The limitation of this study was the cross-sectional nature of the data that could obscure the causal effect relationships of different factors and it lacks qualitative data. Basically, the study addressed the sensitive issue about menstrual hygiene and the possibility of social desirability bias is unavoidable even if we have tried our best to minimize it.

### Conclusions and recommendations

Half of the participants had good knowledge of menstruation and menstrual hygiene. The practice of menstrual hygiene was low (39.9 %). Indeed, the findings showed a significant positive association between good knowledge of menstruation and educational status of the mother, having radio/TV. The educational status of the mother and the earning of permanent pocket money from families or relatives revealed significant positive association with good practice of menstrual hygiene. Awareness regarding the need for information about good menstrual practices is very important. Mass media should also emphasize on health information about menstrual hygiene. Therefore, policy makers and stakeholders should setup health education program to create awareness and practice of good menstrual hygiene (Additional file 1).

## Additional file

Additional file 1:
**Structured Questionnaire (English Version).** (DOCX 49 kb)
