# Supplementary material for: Assessment of knowledge and practice of menstrual hygiene among high school girls in Western Ethiopia
Source: BMC Womens Health. 2015 Oct 14;15:84. doi: 10.1186/s12905-015-0245-7 (PMC4606849; doi:10.1186/s12905-015-0245-7)
Supplement: Additional file 1: — Structured Questionnaire (English Version). (DOCX 49 kb) [file 12905_2015_245_MOESM1_ESM.docx]

## Structured Questionnaire (English Version)

Questionnaire on assessment of knowledge, attitude and practice about menstruation and menstrual hygiene among secondary high school girls at Nekemte town, western Ethiopia, 2014.

**Consent form that certify the respondents agreement before the interview**

01. Name of the School _______________________________________

03. Questionnaire Identification Number__________________________

**Dear Respondent,**

We are interested in learning more about your knowledge, attitude and practice about menstruation and menstrual hygiene. This questionnaire is designed for a research work approved by Wollega University.

We hope you will help us by completing this survey. None of your answers will be available to anyone at any time. All the information you give us will be kept private. Do not put your name anywhere on this questionnaire. If you decide not to participate or complete the form, you may end filling the questionnaire at any time you want However, we really need your honest response to better understand knowledge, attitude and practice about menstruation and menstrual hygiene in high school girl students.

The results of the study would hopefully serve as an important input to intervention programs that aim at improving adolescent girls health in general and students in high school in particular.

It will take you 35-40minutes to complete the whole questionnaire.

We thank you in advance for taking your time to respond to our questions!

Would you be willing to participate in the study? Agree Disagree

If you decide not to participate in the study, please return the questionnaire to the supervisor/investigator.

**Questionnaire Form Identification No _______________________**

Instruction: circle the responses or write the appropriate answer on the space provided.

| **Part I : Socio-Demographic Variables** | | | |
| --- | --- | --- | --- |
| **S.No** | **Questions** | **Option/Response** | **Remark** |
|  | **School** |  |  |
| 101 | Age | __________(in years) |  |
| 102 | Grade | 1. Ninth 2. Tenth |  |
| 103 | Ethnicity | 1. Oromo 2. Amhara 3. Gurage 4. Tigrae 5. Other(Specify )_______________ |  |
| 104 | Religion | 1. Protestant 2. Orthodox 3. Catholic 4. Muslim 5. Others(Specify)_____________ |  |
| 105 | Whom do you live with at present?(More than one answer is possible) | 1. With my mother and father 2. With my mother only 3. With my father only 4. with step mother and my father 5. With step father and my mother 6. With relatives 7. With friends 8. Alone 9. Other(specify) |  |
| 106 | What is your father’s educational level? | 1. Illiterate (cannot read and write) 2. Literate (able to read and write) 3. Primary school – 1- 4 grade 4. Primary school – 5- 8 grade 5. Secondary school 6. College diploma and above |  |
| 107 | What is your mother’s educational level? | 1. Illiterate (cannot read and write) 2. Literate (able to read and write) 3. Primary school – 1- 4 grade 4. Primary school – 5- 8 grade 5. Secondary school 6. College diploma and above |  |
| 108 | Occupation of father | 1. Farmer  2. Government employee  3. Merchant/Trade  4. Private Org. employee  5. Daily laborer  6. Other (specify)_____________ |  |
| 109 | Occupation of mother | 1. Housewife only  2. Farmer  3. Merchant/Trade  4. Private Organization employee  5. Government employee  6. Daily laborer  7. Other (specify)_________________ |  |
| 110 | Do you get permanent pocket money from your parents? | 1. Yes 2. No |  |
| 111 | Does your family have a TV or/and radio? | 1. Yes 2. No |  |
| 112 | Do you earn money for yourself? | 1. Yes 2. No |  |
| 113 | Monthly income of your family | _______________ ETB/Month |  |
| **Part- II About menstruation** | | | |
| 201 | When did you attain your menarche? | ______________ Years.  Don’t Know |  |
| 202 | What was the reaction to your first menstruation? | 1. Happy 2. Scared 3. Discomfort 4. Emotional disturbance 5. Other( specify) :­­­___________ |  |
| 203 | What were your physical symptoms when the first time you had menstruation? | 1. Abdominal and back pain 2. Sleeplessness 3. Heavy bleeding 4. Other(specify) |  |
| 204 | In how many days’ interval was your menses occurring, when you started menstruating? | ____________ days |  |
| 205 | What is the average duration of your menstruation flow? | ____________ days |  |
| 206 | Before the onset of menstruation, have you had any class session related to it in your school? | 1. Yes 2. No |  |
| 207 | If yes, for Q206 what was the subject you learnt on? | _______________ subject |  |
| 208 | Amount of bleeding during menstruation? | 1.Scanty  2. Heavy  3.Moderate |  |
| 209 | Do you have any problem associated with menstruation? | 1. Headache 2. Vomiting 3. Weakness 4. Anorexia 5. Abdominal pain 6. Back pain 7. Other(Specify) |  |
| **Part- III Knowledge on menstruation** | | | |
| 301 | What is menstruation? | 1. Physiological 2. Pathological 3. Curse 4. Other(Specify) ______________ 5. Don’t know |  |
| 302 | What is the cause of menstruation? | 1. Hormones 2. Curse of god 3. Caused by disease 4. Others(Specify)_______________ 5. Don’t know |  |
| 303 | From which organ does menstrual blood comes? | 1. Uterus 2. Vagina 3. Bladder 4. Abdomen 5. Other(Specify)________________ 6. Don’t know |  |
| 304 | At what age, do you think most girls usually get their first period? | ­­­­­_______________ years  Don’t know |  |
| 305 | What is the duration of normal menstruation, in normal person? | ____________ days.  Don’t know |  |
| 306 | What is the interval between two menstrual cycles? (How long is it between one menstrual cycle to the next?) | ____________days.  Don’t know |  |
| 307 | Did anyone tell you about menstruation before you started menstruating? | 1. Yes 2. No |  |
| 308 | From whom you got information regarding about menstruation? (More than one answer is possible) | 1. Mother  2. Teacher  3. Friends  4. Books  5. Media (TV, Radio)  6. Others(Specify)______________ |  |
| 309 | Do you think menstrual problems  interfere with school performance? | 1. Yes 2. No |  |
| 310 | Do you know about menstrual hygiene? | 1. Yes 2. No |  |
| 311 | If yes to Q310, What do you know about menstrual hygiene? (Write in two lines) | ------------------------------------------ |  |
| 312 | Do you think there is foul odor during menstruation? | 1. Yes 2. No |  |
| 313 | Do you think menstrual blood is unhygienic? | 1. Yes 2. No |  |
| **Part-IV: Practices of Menstruation** | | | |
| 401 | Do you use absorbent material during period? | 1. Yes 2. No |  |
| 402 | What absorbent material do you use during menstruation?(More than one answer is possible) | 1. Commercially made sanitary pad 2. Napkin (soft paper) 3. Rag made pad 4. Cloth 5. Other(specify)________________ |  |
| 403 | If you are using cloth as pad How do you clean it? | 1. Soap &water 2. Only water 3. Other (Specify ):______________ |  |
| 404 | If you are using cloth How do you dry the cloth? | 1. Sunlight 2. Inside the house 3. Other(Specify) |  |
| 405 | How many times do you change the cloth/pad in a day? | 1. Three and more 2. Once 3. Twice |  |
| 406 | Where do you dispose your pads? | 1. Dustbin 2. Drain 3. Toilet 4. Open field 5. Other (specify)___________ |  |
| 407 | Types of pads wrap used for disposing it? | 1. Papers 2. Plastic bag 3. Not wrap 4. Other(Specify)________________ |  |
| 408 | Number of changing panty per day | _______________ |  |
| 409 | Do you come to school during menstruation? | 1.Yes  2. No |  |
| 410 | If No, why? | _____________________________ |  |
| 411 | If your menstrual problem interferes with attendance, how often does it do so? | 1. One day every cycle 2. Two days every cycle 3. Three days every cycle 4. Four days every cycle 5. Other(Specify)______________ |  |
| 412 | Do you use any medication for menstrual problems? | 1. Yes If no skip to Q 2. No |  |
| 413 | What is the medication you used? | ________________ |  |
| 414 | When will you bath during  Period? | 1. Daily 2. First day 3. Second day 4. Not take any time 5. Other(Specify) |  |
| 415 | Do you clean your genitalia during menstruation? | 1. Yes  2. No |  |
| 416 | If yes for Q mostly by what? | 1. Water and soap 2. Only with water 3. Tissue paper 4. Towel 5. Other(Specify) |  |
